# TurboID screening of ApxI toxin interactants identifies host proteins involved in *Actinobacillus pleuropneumoniae*-induced apoptosis of immortalized porcine alveolar macrophages

**DOI:** 10.1186/s13567-023-01194-6

**Published:** 2023-07-20

**Authors:** Yaofang Hu, Changsheng Jiang, Yueqiao Zhao, Hua Cao, Jingping Ren, Wei Zeng, Mengjia Zhang, Yongtao Li, Qigai He, Wentao Li

**Affiliations:** 1 National Key Laboratory of Agricultural Microbiology, College of Animal Sciences and Veterinary Medicine, Huazhong Agricultural University, Wuhan, 430070 China; 2 The Cooperative Innovation Center for Sustainable Pig Production, Wuhan, 430070 China; 3 Hubei Hongshan Laboratory, Wuhan, 430070 China; 4 Key Laboratory of Prevention & Control for African Swine Fever and Other Major Pig Diseases, Ministry of Agriculture and Rural Affairs, Wuhan, China; 5 College of Veterinary Medicine, Henan Agricultural University, Zhengzhou, 450002 China

**Keywords:** *Actinobacillus pleuropneumoniae*, ApxI exotoxin, TurboID enzyme-catalyzed proximity labeling method, interacting proteomes

## Abstract

*Actinobacillus pleuropneumoniae* (APP) is a gram-negative pathogenic bacterium responsible for porcine contagious pleuropneumonia (PCP), which can cause porcine necrotizing and hemorrhagic pleuropneumonia. *Actinobacillus pleuropneumoniae*-RTX-toxin (Apx) is an APP virulence factor. APP secretes a total of four Apx toxins, among which, ApxI demonstrates strong hemolytic activity and cytotoxicity, causing lysis of porcine erythrocytes and apoptosis of porcine alveolar macrophages. However, the protein interaction network between this toxin and host cells is still poorly understood. TurboID mediates the biotinylation of endogenous proteins, thereby targeting specific proteins and local proteomes through gene fusion. We applied the TurboID enzyme-catalyzed proximity tagging method to identify and study host proteins in immortalized porcine alveolar macrophage (iPAM) cells that interact with the exotoxin ApxI of APP. His-tagged TurboID-ApxIA and TurboID recombinant proteins were expressed and purified. By mass spectrometry, 318 unique interacting proteins were identified in the TurboID ApxIA-treated group. Among them, only one membrane protein, caveolin-1 (CAV1), was identified. A co-immunoprecipitation assay confirmed that CAV1 can interact with ApxIA. In addition, overexpression and RNA interference experiments revealed that CAV1 was involved in ApxI toxin-induced apoptosis of iPAM cells. This study provided first-hand information about the proteome of iPAM cells interacting with the ApxI toxin of APP through the TurboID proximity labeling system, and identified a new host membrane protein involved in this interaction. These results lay a theoretical foundation for the clinical treatment of PCP.

## Introduction

*Actinobacillus pleuropneumoniae* (APP), which can cause porcine contagious pleuropneumonia (PCP), was first discovered in 1957, and to date, a total of 19 serotypes and two biovars have been identified [1, 2]. There is a wide range of severity of PCP, from acute to chronic, based on the immune status of the host, as well as the type and concentration of bacteria that reach the lungs. It is possible for pigs suffering from acute or peracute diseases to exhibit some/all of the following clinical signs: fever, anorexia, ataxia, vomiting, diarrhea, and severe respiratory distress with cyanosis. Therefore, APP is listed as one of the ten pathogens that have the greatest impact on the pig farming industry [3]. It has been reported that APP exhibits a variety of virulence factors that are grouped into the following categories: adhesion, acquisition of nutrients, induction of lung lesions, evasion of the immune system, and persistence [4, 5]. ApxI-III are among the most important cytotoxic factors produced by APP. There are several types of toxins, but ApxI is one of the most hemolytic and cytotoxic toxins and can be produced by all highly virulent strains, such as serotypes 1, 5, 9 and 11 [6, 7]. The ApxI toxin gene secreted by APP is divided into four CABD parts, where the A gene is the structural gene of the toxin protein, the C gene is the activating gene, and the BD gene is the secretory gene [8]. In a subsequent study, we expressed ApxIA, the structural gene fragment. It has previously been demonstrated that low concentrations of ApxI toxin can induce apoptosis in porcine alveolar macrophages [9]. There seems to be a lack of understanding of the role of ApxI toxin in apoptogenic porcine alveolar macrophages. Using TurboID-mediated proximity labeling, this study aimed to identify the host proteins that interact with ApxI in porcine alveolar macrophages and to determine the role of ApxI in macrophage apoptosis.

Protein interactions are the basis for many biological processes. In recent years, an innovative approach has emerged to study the spatial and interaction characteristics of proteins in living organisms: enzyme-catalyzed proximity labeling (PL). The most widely used neighboring labeling enzymes for living cells and organisms are engineered ascorbate peroxidase 2 (APEX2) [10] or biotin ligases (BioID [11], BioID2 [12], BASU [13], TurboID [14], miniTurbo [14]). Among them, a new labeling enzyme called TurboID, a mutant of the *E. coli* biotin ligase BirA, was developed to label proteins of interest in just 10 min [14]. There are relatively few studies using TurboID to study the interaction of bacterial virulence factors with the host. A recent study was conducted on *Glaesserella parasuis* (GPS) interactions with host cells and identified three membrane proteins related to GPS adhesion and invasion [15].

To identify cellular proteins that interact with ApxI at the surface of alveolar macrophages, we utilized TurboID enzyme-catalyzed proximity labeling. Specific interacting proteins were identified by mass spectrometry, membrane proteins were identified using the UniProt website, and their interaction with ApxI was confirmed by co-immunoprecipitation (Co-IP) analysis. Subsequently, we demonstrated that caveolin-1 (CAV1) protein is required for ApxI-induced apoptosis of porcine alveolar macrophages by overexpression and RNA interference experiments. Finally, we constructed ApxIA truncated proteins for the Co-IP assay to confirm the specific interaction between ApxI toxin and CAV1 protein.

## Materials and methods

### Bacterial strains and culture conditions

*A. pleuropneumoniae* serotype 1 (strain 4074) and serotype 10 (strain 13039) are conserved in our laboratory. Both serotype strains secrete at least one exotoxin, including ApxI toxin. *A. pleuropneumoniae* serotypes 1 and 10 were cultured in tryptic soy broth (TSB) medium or tryptic soy agar (TSA) plates supplemented with 10 μg/mL NAD at 37 °C. *Escherichia coli* DH5α and *Escherichia coli* BL21 (DE3) were cultured in TSA plates or Luria–Bertani (LB) medium at 37 °C.

### Cells and culture conditions

The immortalized swine pulmonary alveolar macrophage (iPAM) cell line and human embryonic kidney 293 T (HEK 293 T) cell line are conserved in our laboratory. The iPAM cells were cultured and maintained in RPMI 1640 medium with 10% FBS. HEK 293 T cells were cultured and maintained in DMEM with 8% FBS.

### Plasmids

The pcDNA-TurboID plasmid was conserved in our laboratory. To generate the His-TurboID-ApxIA fusion construct, using the pcDNA-TurboID plasmid and *A. pleuropneumoniae* 1 genomes as templates (NCBI Reference Sequence: NZ_CP030753.1), TurboID and ApxIA fragments were amplified by PCR using primers P1/P2 and P3/P4 (Phanta Super-Fidelity DNA Polymerase, Vazyme, China). These two fragments were linked with overlap extension PCR to construct TurboID-ApxIA and then inserted into the pET-28a plasmid with NheI and XhoI restriction enzymes to generate the recombinant expression plasmid pET-28a-TurboID-ApxIA. His-TurboID was amplified using primers P5/P6 from pcDNA-TurboID and cloned into the pET-28a plasmid with NheI and XhoI restriction enzymes to generate the recombinant expression plasmid pET-28a-TurboID. The two recombinant plasmids were confirmed by sequencing.

The pET-28a-HA-His plasmid, which could express both HA Tag and His Tag, was conserved in our laboratory. HA-ApxIA was amplified using primers P7/P8 from the APP1 genome and cloned into the pET-28a-HA-His plasmid with NdeI and XhoI restriction enzymes to generate plasmid pET-28a-His-ApxIA.

The CDS of porcine CAV1 was amplified from iPAM cells by RT‒PCR using primers P9/P10 and cloned into vector pCAGGS-Flag with EcoRI and XhoI restriction enzymes to generate the expression plasmid Flag-CAV1. The plasmids were verified by sequencing. The primers used in this study are listed in Table 1.

**Table 1:** **T﻿he primers used to construct plasmids.**

Primers	Sequence	Characteristics
P1	CTAGCTAGCGGTGGCAGCGGTGGCAGCATGAAAGACAATACTGTGCC	To amplify the TurboID fragment
P2	GCTACTAGTGTTTTTTTCATGCTGCCACCGCTGCCACCCTTTTCGGCAGACCGCAGAC	
P3	GGAATTCCATAATGGGTGGCAGCGGTGGCAGCATGGCTAACTCTCAGCTCG	To amplify the ApxIA fragment
P4	CCGCTCGAGAGCTGCTTGTGCTAAAGAATAACTCAAAGAA	
P5	CTAGCTAGCGGTGGCAGCGGTGGCAGCATGAAAGACAATACTGTGCC	To amplify the His- TurboID-fragment
P6	CCGCTCGAGCTTTTCGGCAGACCGCAGACTG	
P7	GGAATTCCATAATGGGTGGCAGCGGTGGCAGCATGGCTAACTCTCAGCTCG	To amplify the HA-His- ApxIA fragment
P8	CCGCTCGAGAGCTGCTTGTGCTAAAGAATAACTCAAAGAA	
P9	CCGGAATTCGGTGGCAGCGGTGGCAGCATGTCGGGGGGCAAATACGTAG	To amplify CAV1 gene
P10	CCGCTCGAGTTATATTTCTTTCTGCATG	
P11	CGCCATATGGGTGGCAGCGGTGGCAGCGCTGCAACCGGCTCATTAG	To amplify the HA-His-ApxI-M1 fragment
P12	CCGCTCGAGAGCTGCTTGTGCTAAAGAATAAC	To amplify the HA-His-ApxI-M1, HA-His-ApxI-M2, and HA-His-ApxI-M3 fragment
P13	CGCCATATGGGTGGCAGCGGTGGCAGCGGACAAAGTGCACAGAAAGC	To amplify the HA-His-ApxI-M2 fragment
P14	CGCCATATGGGTGGCAGCGGTGGCAGCAACTTATTTGACGGTGGTGTAG	To amplify the HA-His-ApxI-M3 fragment
P15	CGCCATATGGGTGGCAGCGGTGGCAGCATGGCTAACTCTCAGCTCGA	To amplify the HA-His-ApxI-M4 fragment
P16	CCGCTCGAGGTCAAATGTTAAGTAACCTGTATCGG	

Four truncated ApxIA protein expression constructs, pET-28a-His-ApxI-M1, pET-28a-His-ApxI-M2, pET-28a-His-ApxI-M3, and pET-28a-His-ApxI-M4, were generated from pET-28a-His-ApxIA using primers P11/P12, P13/P12, P14/P12, and P15/P16, respectively. The quality of the plasmids was confirmed by sequencing.

### Expression of His-TurbolD-ApxIA, His-TurboID, HA-His-ApxIA and truncated proteins

The plasmids pET-28a-TurboID-ApxIA, pET-28a-TurboID, pET-28a-HA-His-ApxIA, pET-28a-His-ApxI-M1, pET-28a-His-ApxI-M2, pET-28a-His-ApxI-M3, and pET-28a-His-ApxI-M4 were transformed into *E. coli* BL21 (DE3) for recombinant protein expression. Transformants were cultured in LB medium containing 50 μg/mL kanamycin at 37 °C for 16 h, transferred into fresh LB medium containing 50 μg/mL kanamycin at a ratio of 1:10 and allowed to grow at 37 °C with shaking until the OD_600nm_ reached 0.6 IPTG (0.5 mM) was then added to the cultures to induce recombinant protein expression.

The IPTG-induced bacteria were pelleted at 8000 rpm for 20 min at 4 °C and resuspended in ice-cold resuspension buffer (1 M Tri-HCl pH 7.4, 0.5 M NaCl, 20 mM imidazole, protease inhibitor cocktail) and disrupted thrice under 25 kpsi by French press (Constant system) at 4 ℃. Crude extracts were collected by centrifugation at 12 000 rpm for 15 min at 4 °C. After centrifugation, the supernatant was filtered through a 0.22 μM membrane to remove any debris.

The obtained supernatant was loaded on a His Sep Ni–NTA Agarose Resin (YEASEN, Shanghai, CHN) column preequilibrated with 10 column volumes of ice-cold buffer A (1 M Tri-HC1, 0.5 M NaCl, pH 7.4). Filtrated crude extracts were subsequently loaded onto the column at a flow rate of 0.5 mL/min at 4 °C, and the protein-bound resin was washed with PBS (pH 7.4) containing 20 mM imidazole at a flow rate of 1 mL/min. Buffer B in this step contained the same buffering species but with a higher imidazole concentration (0.5 M imidazole). After the sample was loaded onto the column, proteins were eluted by a 15 min linear gradient elution (buffer composition was changed from 0% of buffer B to 100% of buffer B within 15 min). SDS‒PAGE and Coomassie Blue staining were then used to detect the purity of each collected fraction. The fractions that contained pure recombinant proteins were pooled accordingly and subsequently concentrated. The collected proteins were packed into treated dialysis bags, sealed tightly with clamps at both ends of the bags, and the bags were placed in a flat dish with PEG 4000 for 2–4 h for reverse dialysis concentration, changing PEG 4000 several times in process, and when concentrated to 1/5 of the original volume, the proteins were removed from the dialysis bags and stored at − 70 °C.

### Biotin and streptavidin affinity purification

iPAM cells were seeded into 6-well tissue culture plates in RPMI 1640 medium containing 10% FBS for 24 h. After the cells had grown to 100% confluence, the cells were washed three times with sterile PBS, and 16 μg His-TurboID-ApxIA or His-TurboID recombinant protein was added to fresh RPMI 1640 medium and incubated on ice for 1 h to allow the proteins to be recognized by iPAM cells. Then, 50 μM biotin (Sigma) was added and incubated at 37 °C for 20 min to label the interacting proteins. The reaction was then quenched, and the cells were washed three times by removing the suspension liquid and replacing it with ice-cold PBS. Cells (two wells per sample) were then lysed in 500 μL lysis buffer (Cell lysis buffer for Western and IP, Beyotime, China) containing protease inhibitors. Residual cells and cell debris adhering to the culture plates were scraped into the lysates, incubated on an end-over-end rotator at 4 °C for 1 h, and centrifuged at 14 000 × *g* for 10 min at 4 °C. Supernatants were incubated with streptavidin magnetic beads on a rotator at 4 °C overnight (250 μL beads per sample, NEB, USA) that were previously washed with binding buffer (20 mM Tri-HCl pH 7.5, 0.5 M NaCl, 1 mM EDTA). Then, the beads were washed three times with cell lysis buffer. Biotinylated proteins were then eluted by boiling the beads in 100 μL SDT buffer (4% (m/v) SDS, 100 mM Tris–HCl, 1 mM DTT, pH 7.6), followed by mass spectrometry or SDS‒PAGE analysis.

### Mass spectrometry analysis

The samples were analyzed by mass spectrometry in the Omics Space (Shanghai, China). The acquired data from triplicate MS runs for each sample were combined and searched against uniprot_Sus_scrofa_333904_20190905. Fasta protein sequence database using the MaxQuant computational proteomics platform version 2.0.1.0. Proteins were identified using the Andromeda peptide search engine integrated into the MaxQuant environment. A decoy version of the Self-database was used to estimate peptide and protein false discovery rates. The maximum protein and PSM false discovery rates were set to 0.01. Carbamidomethylation of cysteine was set as a fixed modification, with protein oxidation of methionine as a variable modification, enzyme: trypsin/P, maximum number of missed cleavages: 2. We used MaxQuant to calculate LFQ, a measure of protein abundance. The LFQ value is obtained by dividing protein intensities by the number of theoretically observable tryptic peptides between 5 and 30 amino acids and is on average highly correlated with protein abundance. The Retrieve/ID Mapping tool was utilized for subcellular location designations of identified candidate proteins.

### Overexpression and RNA interference experiments

For the overexpression assay, 5 × 10^5^ iPAM cells were seeded into 24-well tissue culture plates in RPMI 1640 medium containing 10% FBS. At 60%-70% confluence, cells were transfected with the Flag-CAVl expression plasmid and Flag-vector control using jetPRIME transfection reagent (Polyplus Transfection) for 24 h, according to the manufacturers’ instructions. For siRNA-mediated knockdown, 5 × 10^5^ iPAM cells were seeded into 24-well tissue culture plates in RPMI 1640 medium containing 10% FBS. After the cells had grown to 60–70% confluence, they were transfected with 50 nM CAV1-targeting siRNAs or negative control siRNA using jetPRIME transfection reagent as recommended by the manufacturer for 24 h. Double-stranded siRNAs targeting CAV1 and negative control were designed and synthesized by Genepharma Co. (Shanghai, China) (Table 2).

**Table 2:** **The sequence of siRNA used in this study.**

Gene		siRNA sequence (5ʹ–3ʹ)
CAV1	Sense	GGAAAUGAACGAGAAGCAATT
	Antisense	UUGCUUCUCGUUCAUUUCCTT
Negative control	Sense	UUCUCCGAACGUGUCACGUTT
	Antisense	ACGUGACACGUUCGGAGAATT

### Coimmunoprecipitation (Co-IP) assay

To investigate the interaction of the CAV1 protein with ApxI toxin, HEK 293 T cells, which are insensitive to ApxI, were used for transfection. HEK 293 T cells seeded into 6-well culture plates were transfected with the corresponding expression plasmids. Transfected cells were harvested at 48 h and lysed in cell lysis buffer containing 1 mM protease inhibitor. After centrifugation at 12 000 × *g* for 10 min, the lysate supernatants containing 1–2 mg of total protein were incubated with 2.5 μg HA-ApxIA or truncated ApxIA proteins for 4 h with gentle rocking at 4 °C and then incubated overnight with mouse monoclonal antibody against Flag, His or HA tag with gentle rocking at 4 °C. Protein A/G beads washed with cell lysate were added to the supernatants and incubated with gentle rocking for 4 h at 4 °C. Beads were washed four times with cold cell lysate and boiled with 1 × SDS loading buffer for 10 min, followed by western blotting.

### Preparation of *Actinobacillus pleuropneumoniae* exotoxin ApxI

APP serotype 10 (strain 13039) is kept in our laboratory. This strain secretes only ApxI toxin under in vitro culture conditions. It is often used to extract active toxins. The exotoxin of *A. pleuropneumoniae* serotype 10 was prepared as a previously described method with some minor modifications [16]. Cells were grown to the mid-log phase in PPLO medium containing 10 µg/mL NAD and 20 mM CaCl_2_. The culture supernatants were collected using centrifugation at 8000 × *g* for 30 min at 4 °C and mixed with 55% ammonium sulfate, followed by incubation at 4 °C overnight. The precipitated toxins were pelleted at 17 000 × *g* for 30 min at 4 °C, dissolved in 10 mM Tris–HCl (pH 7.5) at a ratio of 1/85 of the original culture volume, and dialyzed in the same buffer at 4 °C for 48 h. The collected toxins were stored at − 70 °C until use.

The extracted toxin proteins were identified by western blotting using mouse polyclonal antibodies kept in our laboratory. The concentration of toxin proteins is determined using ultra-micro spectrophotometer (Biospec-nano, Japan). A hemolytic activity test was carried out to determine if the extracted toxin ApxI was active. The hemolysis assay was performed according to a previously described method with minor modifications [17]. Briefly, ApxI toxin was serially diluted 2 times with 0.9% NaCl solution and incubated with washed red blood cells for 2 h at 37 °C. The toxin diluted in multiples is capable of lysing different proportions of porcine red blood cells. The percentage of hemolysis was calculated by measuring the absorbance at 550 nm using a plate reader (PerkinElmer VICTOR Nivo, USA).

### Apoptosis assay

The assays were performed based on a previously described method with some minor modifications [9]. iPAM cells were overexpressed or subjected to siRNA interference as described above and then washed three times with sterile PBS. Cells were stimulated with ApxI toxins at protein concentrations of 50 μg/mL at 37 °C for 34 h.

### Annexin V-FITC/propidium iodide staining

Apoptosis detection was performed using a FITC Annexin V Apoptosis Detection Kit (Beyotime). After exposure to ApxI toxin, cells from each group were washed with PBS to remove any debris. Then, 195 μL annexin V incubation conjugate was added to each sample and gently spread to ensure that the samples were covered. Then, 5 μL Annexin V-FITC and 10 μL propidium iodide staining solution were added and mixed gently. After incubation at RT in the dark for 15 min, fluorescence microscopy (Olympus IX73, Tokyo, Japan) was used to monitor the cells.

### Western blot

The cells were collected and then lysed in a buffer containing 1% Triton X-100, 20 mM Tris–HCl, 137 mM NaCl, 2 mM EDTA, 10% glycerol, 10 mM leupeptin, 1 mM aprotinin, 2 mM benzamidine, 0.5 mM DTT, and 0.5 mM PMSF, pH 7.4. The proteins were isolated by 12% SDS‒PAGE and then electrophoretically transferred onto polyvinyl difluoride (PVDF) membranes (Millipore). PVDF membranes were blocked with 5% bovine serum albumin (BSA) at room temperature for 2 h and then incubated with the respective primary antibodies overnight at 4 °C. After washing three times with TBST, the membrane was incubated with HRP-linked goat anti-rabbit or HRP-linked goat anti-mouse antibody at room temperature for 2 h and visualized using an Omni-ECLTM Femto Light Chemiluminescence Kit (Epizyme Biotech, China). The antibodies used in this study are listed in Table 3. Immunoblots were reprobed with an anti-β-actin antibody as a loading control. The intensity of active caspase 3 was quantified using ImageJ software and normalized to the intensity of the loading control β-actin.

**Table 3:** **Antibodies used in this study.**

Antibody	Name	Supplier	Catalog no
CAV1	Caveolin-1 rabbit monoclonal antibody	Beyotime	AF1231
Caspase-3	Caspase-3 antibody	Beyotime	AC030
β-actin	β-actin rabbit monoclonal antibody (High Dilution)	ABclonal	AC026
ApxI	ApxI toxin polyclonal antibody	Prepared and stored in our laboratory	
Flag	DYKDDDDK tag mouse monoclonal antibody	Proteintech	66008-4-Ig
HA	HA tag mouse monoclonal antibody	Proteintech	66006-2-Ig
Secondary antibody	HRP goat anti-mouse IgG (H + L)	ABclonal	AS003
Secondary antibody	HRP goat anti-rabbit IgG (H + L)	ABclonal	AS014
Streptavidin	Horseradish peroxidase conjugated streptavidin	Sigma	RABHRP3
His-tag	HRP-conjugated 6*His, His-tag monoclonal antibody	Proteintech	HRP-66005
Secondary antibody	HRP goat anti-mouse IgG HCS	Abbkine	A25112
	HRP goat anti-mouse IgG LCS	Abbkine	A25012

### Statistical analysis

Results are presented as the mean ± standard deviation (SD). The results were evaluated by multiple t tests in Graph Pad Prism 7.0 (Graph Pad Software Inc., USA). *P* < 0.05 was considered statistically significant (*), while *p* < 0.01 was regarded as highly significant (**) and (***) designated as *p* < 0.001.

## Results

### Expression of His-TurboID-ApxIA and His-TurbolD and validation of their efficiency of biotinylation

**Figure 1:**
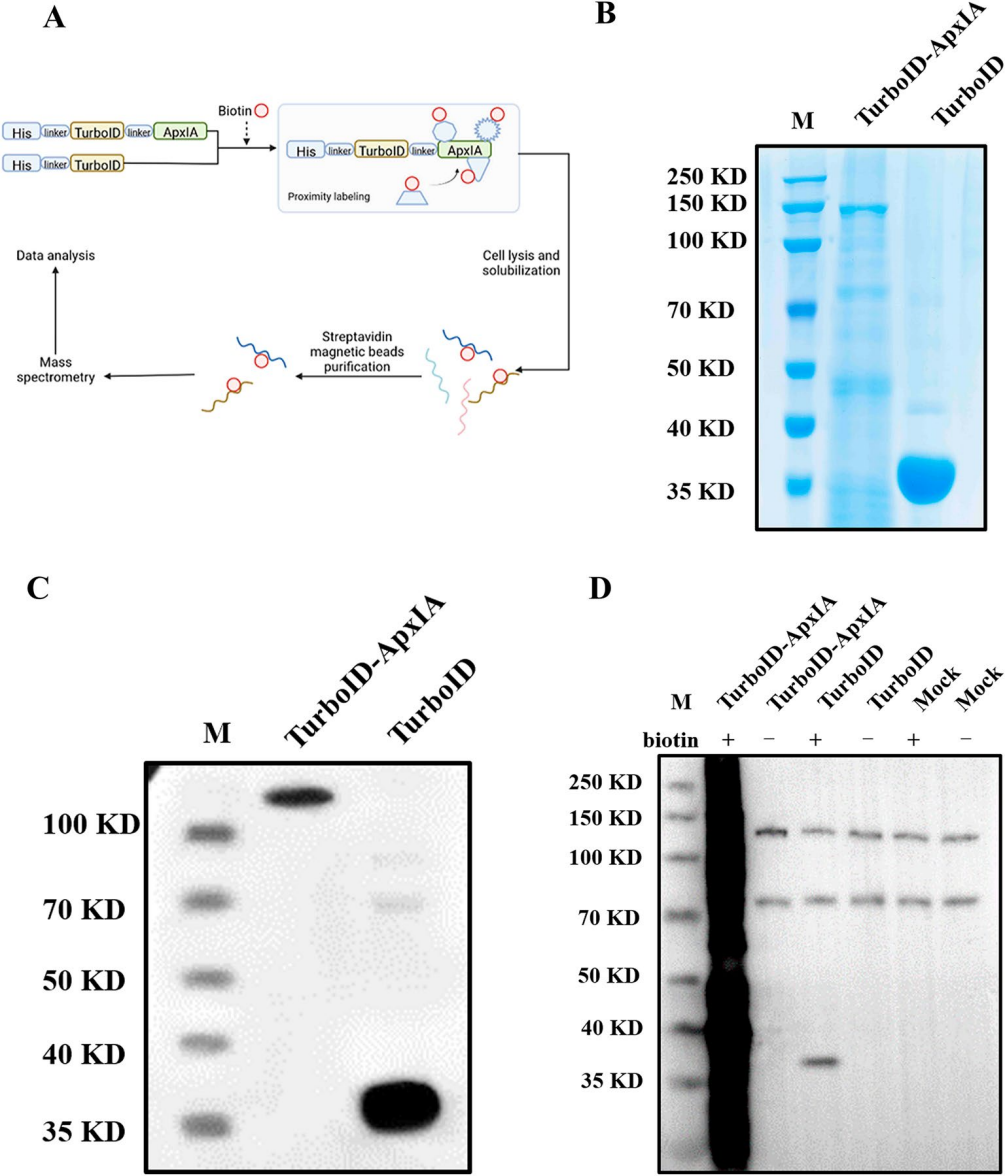
**Expression of His-TurboID-ApxIA and His-TurboID and validation of their efficiency of biotinylation. A** A schematic drawing of the His-TurboID-ApxIA and His-TurboID constructs. The ApxIA gene was ligated to the 3′ end of TurboID with a linker encoding a short peptide (GGSGGS). His tag sequence was added to the 5′ end of TurboID for detection and purification of the recombinant proteins. **B** Analysis of His-TurboID-ApxIA and His-TurboID recombinant protein expression by SDS‒PAGE. **C** Analysis of His-TurboID-ApxIA and His-TurboID recombinant protein expression by western blot. **D** Western blot analysis of His-TurboID-ApxIA- and His-TurboID-treated iPAM cells. iPAM cells were treated with His-TurboID-ApxIA, His-TurboID, or untreated in medium with and without supplementation with 50 μM biotin. Total cell lysates were detected by western blot probed with horseradish peroxidase (HRP)-coupled streptavidin.

The ApxIA gene was linked to the TurboID gene with a six amino acid GS linker (GGSGGS) in the expression vector pET-28a, and a His-tag was fused to the N terminus of TurboID to create a fusion protein for proximal biotin labeling (Figure 1A). His-TurboID served as a control. The fusion proteins His-TurboID-ApxIA and His-TurboID were identified by SDS‒PAGE and western blotting after the expression plasmids His-TurboID-ApxIA and His-TurboID were transformed into *E. coli* BL21(DE3). Recombinant proteins His-TurboID-ApxIA and His-TurboID were verified by SDS‒PAGE and western blot (Figure 1B and C).

We used western blot analysis to further assess the effectiveness and specificity of TurboID-mediated biotinylation. The biotinylated protein fractions were taken from His-TurboID-ApxIA, His-TurboID, or mock-treated iPAM cells with or without free biotin in the culture medium. Endogenously biotinylated proteins were observed in all groups. The same result was detected in iPAM cells when the culture medium was with or without 50 μM biotin, indicating that the addition of biotin in the absence of the TurboID biotin ligase does not significantly change the proportion of endogenously biotinylated proteins. The lysates derived from iPAM cells treated with His-TurboID-ApxIA or His-TurboID in the presence of free biotin, however, showed a significant increase in the amount of biotinylated proteins. Furthermore, His-TurboID-ApxIA fusion protein-treated cells contained a high proportion of biotinylated proteins (Figure 1D). Based on these results, both His-TurboID-ApxIA and His-TurbolD were capable of efficiently biotinylating target proteins in the presence of free biotin.

### Determination of the APP ApxI-proximal proteome

**Figure 2:**
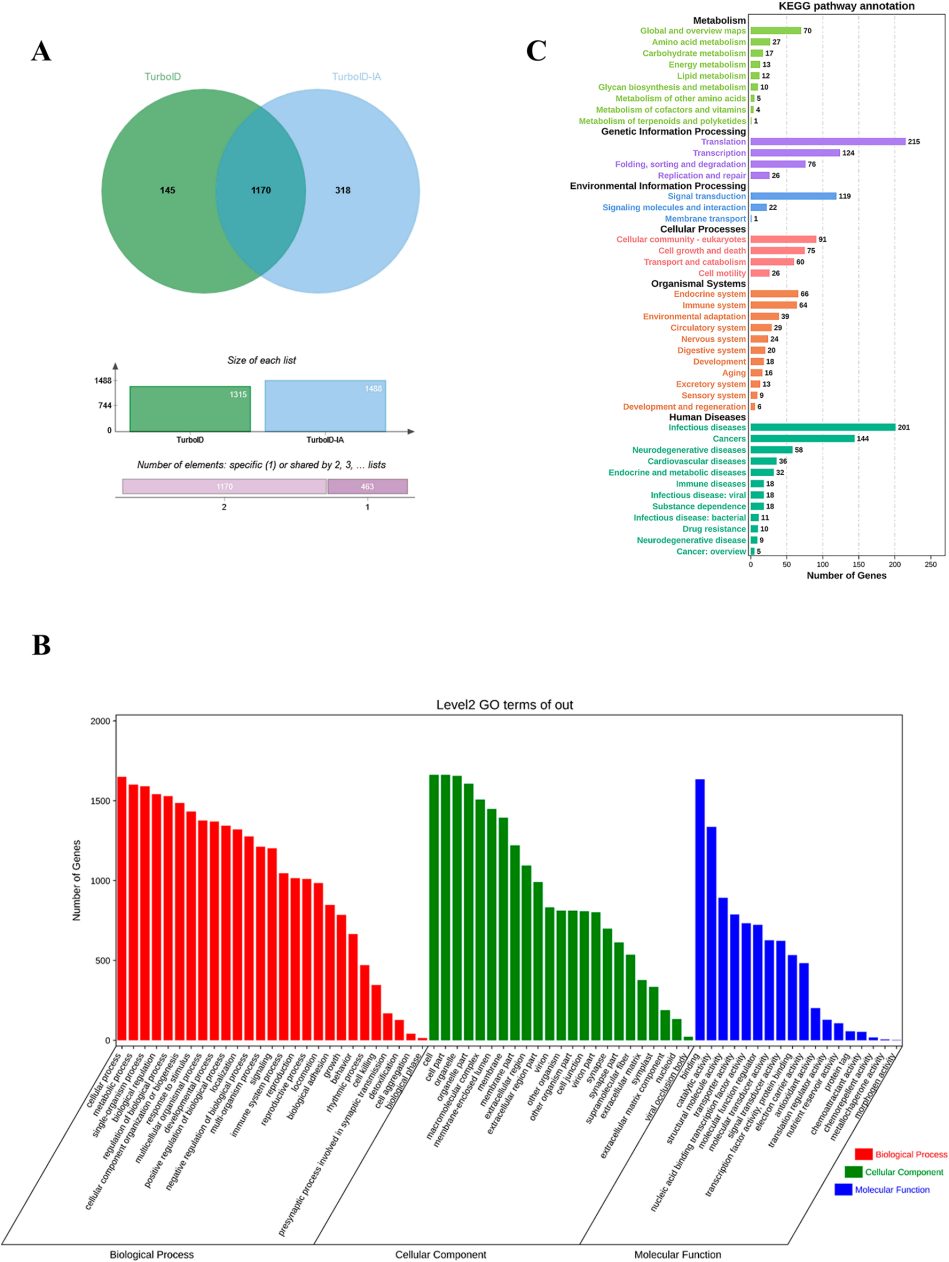
**Bioinformatics analysis of host cell proteins putatively identified as interacting with TurboID-ApxIA proteins. A** Venn diagram of TurboID-ApxIA interacting host proteins and TurboID interacting host proteins. **B** Molecular function of host proteins interacting with ApxIA protein using Gene Ontology. **C** KEGG analysis of host cell proteins interacting with ApxIA protein.

Mass spectrometric analysis of affinity-purified proteins derived from biotin-treated His-TurboID-ApxIA cells and His-TurboID-treated iPAM cells was carried out. According to the results, a total of 1488 and 1315 biotinylated host cell proteins interacted with His-TurboID-ApxIA and His-TurboID, respectively. The His-turboID-ApxIA group contained 318 unique interacting proteins (Figure 2A). Bioinformatics analysis was conducted on all 318 proteins identified. Annotations from the Gene Ontology Consortium website were divided into three main categories: biological processes, cellular components, and molecular functions. The biological process annotation showed that some proteins were involved in the cellular process, cellular component organization or biogenesis, metabolic process, biological regulation, and response to stimulus. Cellular component annotation assigned other proteins to organelle, organelle part, macromolecular complex, membrane, and membrane part. Enrichments based on molecular function annotation were binding, catalytic activity, structural molecule activity, and transporter activity (Figure 2B). The KEGG reference pathway database assigns these 318 sequences to six classes of biometabolic pathways, such as cellular processes, environmental information processing, and genetic information processing (Figure 2C). The aim of this study was to understand the mechanism of interaction between alveolar macrophages and Apx exotoxins. Membrane proteins are often involved in the recognition and phagocytosis of pathogens by iPAM cells and are therefore of particular interest to us. After using the UniProt website and removing the non-membrane proteins, only one membrane protein, CAV1, was identified from these 318 host proteins. Therefore, the function of CAV1 in the interaction between alveolar macrophages and ApxI exotoxins was further investigated.

### Further validation of interactions between ApxI protein and host cell protein

**Figure 3:**
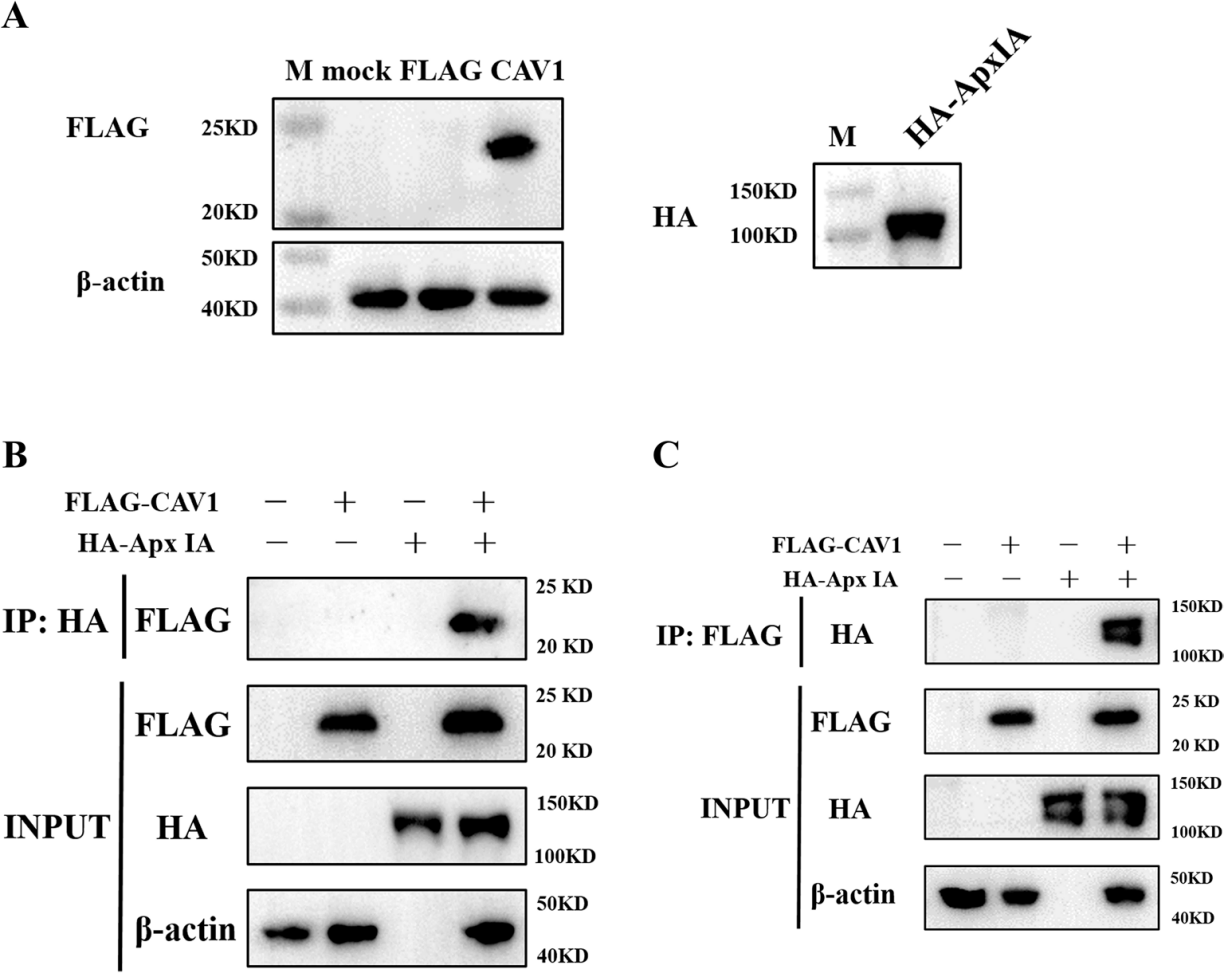
**Validation of interactions between ApxIA protein and host cell proteins with coimmunoprecipitation analysis. A** Western blot analysis of Flag-CAV1 plasmids transfected into HEK 293 T cells. Western blot analysis of pet-28a-HA-His-ApxIA transformed into E.coli BL21(DE3). **B** Immunoblot of Flag-CAV1 recombinant proteins from transfected HEK 293 T cells and HA-ApxIA protein using anti-HA mAb. **C** Immunoblot of HA-ApxIA protein and host cell proteins precipitated using anti-Flag mAb from HEK 293 T cells transfected with pCAGGS-Flag-CAV1.

The Co-IP technique was used to provide further evidence of the interaction between the CAV1 protein of the host cell and the ApxI protein of APP. Overexpression of Flag-CAV1 recombinant proteins was achieved by transfecting HEK 293 T cells with expression plasmids. To express the HA-His-ApxIA fusion protein, the HA-His-ApxIA plasmid was transformed into *E. coli* BL21(DE3). The expression of these constructs was confirmed by western blotting (Figure 3A). Additionally, protein complexes were captured by Co-IP using an anti-Flag mAb or an anti-HA mAb. Based on Figure 3, ApxIA and CAV1 are the only bands present in the same reaction, which can be observed in the IP samples (Figure 3B and C). In conclusion, these results suggest that CAV1 could interact with ApxIA. Therefore, we speculate that CAV1 plays a role in the ApxI protein interaction with iPAM cells.

#### Confirmation of ApxI toxin activity

**Figure 4:**
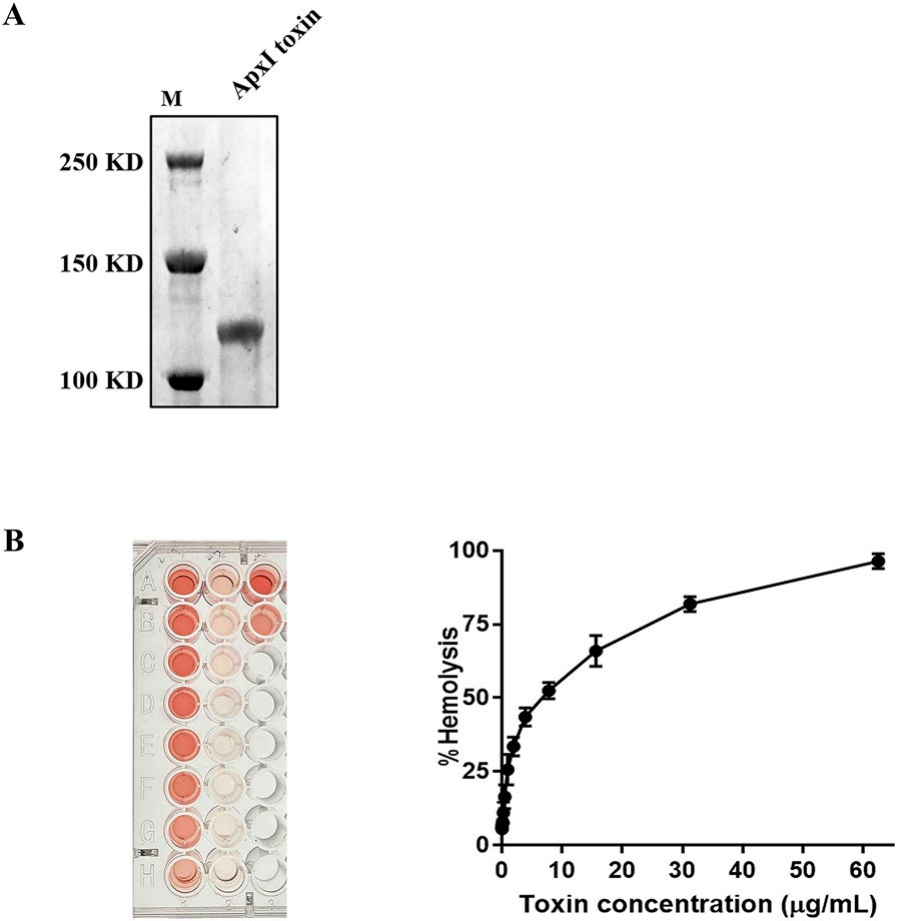
**Identification of the activity of the extracted ApxI toxin**. **A** Analysis of extracted ApxI toxin protein by western blot. **B** Hemolysis test with gradient dilution of ApxI toxin in pig erythrocytes. The results are from three independent experiments of at least duplicate determinations. All data are expressed as the means ± SD.

The expression of extracted ApxI toxin was confirmed by western blotting (Figure 4A). The concentration of the extracted toxin protein was determined to be 0.32 mg/mL. Activity of purified ApxI toxin was assessed by hemolytic test. ApxI toxin (0.24 to 62.5 μg/mL) caused 10 to 100% of the RBCs to undergo hemolysis (Figure 4B and C). Based on these findings, it can be concluded that the purified ApxI toxin is indeed active and can be further subjected to subsequent validation experiments.

### Overexpression of CAV1 promoted ApxI-mediated apoptosis in iPAM cells

**Figure 5:**
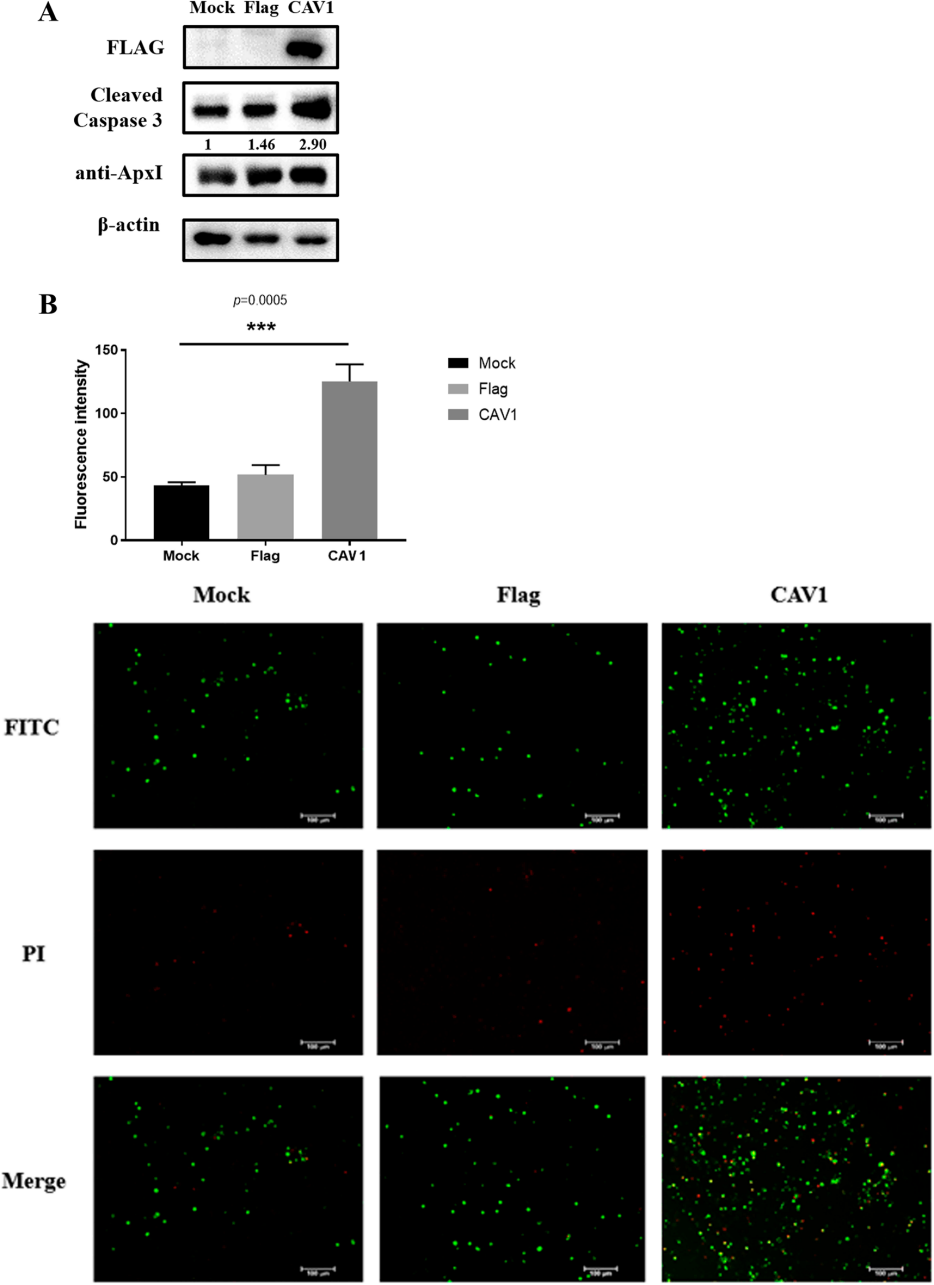
**CAV1 is involved in ApxI-induced apoptosis of iPAM cells. A** Expression plasmid transfection and western blot to assess the protein level of CAV1. Corresponding expression plasmids were transfected into iPAM cells for 24 h. Lysates were separated by SDS‒PAGE and detected by western blotting with an antibody against Flag. Western blot detection of the cleaved caspase 3 band (17 kDa) with a caspase 3 antibody. The average intensity of active caspase 3 in the immunoblot was quantified and normalized to the intensity of β-actin. **B** The effect of CAV1 gene overexpression on apoptosis involving ApxI toxin was examined using the Annexin-V-FITC Apoptosis Detection Kit under fluorescence microscopy. iPAM cells transfected with or without the CAV1 gene were exposed to 50 μg/mL ApxI toxin for 34 h. Graphs show the mean cell apoptosis for each group. A t test showed highly significant changes between the control and overexpression groups (*p* = 0.0005) (*p* < 0.001).

ApxI has a strong cytotoxic effect and can induce apoptosis in porcine cells. To investigate the potential role of CAV1 in ApxI-mediated apoptosis in iPAM cells, western blot experiments were conducted to evaluate the efficiency of CAV1 overexpression. The results showed that CAV1 protein could be overexpressed (Figure 5A). Compared with the control group, cleaved caspase 3 in iPAM cells treated with 50 μg/mL ApxI and CAV1 transfection significantly increased (Figure 5A). Moreover, morphological apoptosis was evaluated to reconfirm the apoptosis of overexpressing cells. A significant increase (*p* = 0.0005) in the number of apoptotic cells was observed in CAV1-vector transfected cells under fluorescence microscopy (Figure 5B). As a result of these findings, it appears that CAV1 promotes the apoptosis of iPAM cells induced by ApxI.

### Knockdown of CAV1 gene expression inhibited ApxI-mediated apoptosis of iPAM cells

**Figure 6:**
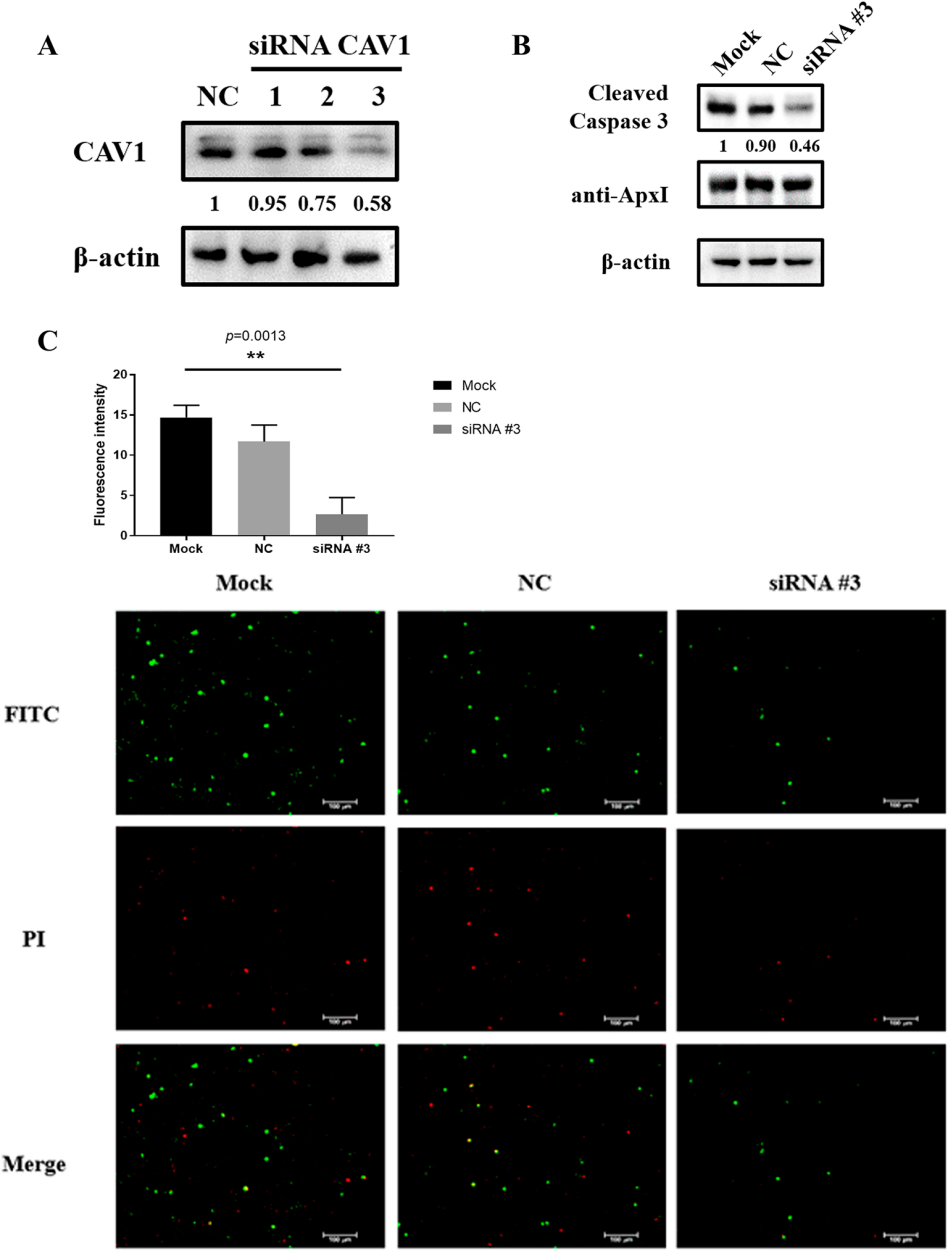
**Knockdown of CAV1 affects apoptosis of iPAM cells by ApxI protein. A** CAV1 protein levels were assessed by siRNA silencing and western blotting. A single 50 nM target-specific siRNA was transfected into iPAM cells for 24 h. Lysates were separated by SDS‒PAGE and detected by western blotting with an antibody against CAV1. **B** Western blot detection of the activated caspase 3 band (17 kDa) with a caspase 3 antibody. The average intensity of active caspase 3 in the immunoblot was quantified and normalized to the intensity of β-actin. **C** The effect of CAV1 gene silencing on ApxI toxin-involved apoptosis was examined under fluorescence microscopy using the Annexin-V-FITC Apoptosis Assay Kit. iPAM cells transfected with or without CAV1 siRNA were exposed to 50 μg/mL ApxI toxin for 34 h. Graphs show the mean cell apoptosis for each group. A t test showed highly significant changes between the control and knockdown groups (*p* = 0.0013) (*p* < 0.01).

To confirm the involvement of CAV1 in ApxI-mediated apoptosis in iPAM cells, siRNA interference experiments were conducted. First, western blots were performed to determine whether siRNA knockdown was effective against CAV1. The results indicated that siRNAs were effective in knocking down target genes, with siRNA CAV1 #3 exhibiting the strongest interference ability (Figure 6A). In western blot experiments, ApxI-mediated apoptosis in CAV1 gene knockdown cells was significantly decreased compared with that in control cells (Figure 6B). Furthermore, a highly significant reduction (*p* = 0.0013) in the number of apoptotic cells was observed in the CAV1 knockout cells compared to the control group (Figure 6C). These results suggested that CAV1 plays an important role in ApxI-mediated apoptosis of iPAM cells.

### ApxI interacts with the CAV1 protein in the acylation segment

**Figure 7:**
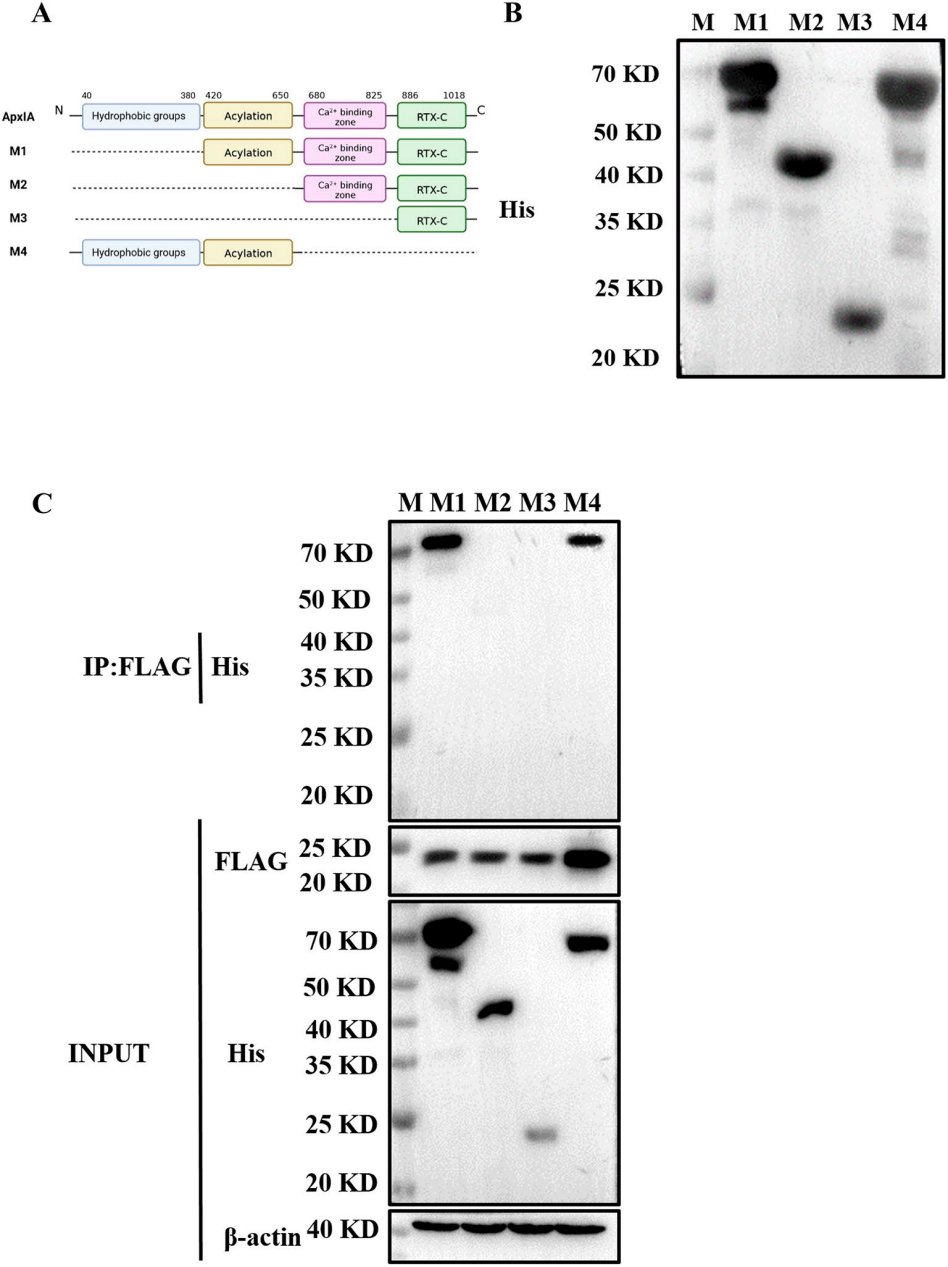
**The acylated structural domain of ApxIA is sufficient to interact with CAV1. A** Schematic representation of the predicted structure and truncated protein of the ApxIA protein of the APP standard reference strain. **B** Immunoblotting of M1, M2, M3 and M4 purified by expression using HRP-His antibody. **C** Detection of Co-IP results using immunoblotting.

We confirmed that the ApxIA protein of APP could interact with CAV1. Furthermore, we demonstrated that CAV1 is involved in ApxI toxin-induced apoptosis in porcine alveolar macrophages. To further identify the key domain(s) of ApxI that interacted with CAV1, four truncated ApxI proteins were expressed and purified. The protein structure of ApxI was predicted by using the Protein Structure Prediction website and based on the results of Seah’s study [18]. As shown in Figure 7A, ApxIA has four functional regions. Based on this predicted structure, we constructed four truncated ApxIA proteins, including M1 (including the acylation region, Ca^2+^ binding region and RTX-C), M2 (including the Ca^2+^ binding region and RTX-C), M3 (RTX-C) and M4 (including the hydrophobic region, acylation region and Ca^2+^ binding region). We expressed and purified four proteins for the Co-IP assay to detect their interactions with CAV1 (Figure 7B). As shown in Figure 7C, M1 and M4 truncates of the ApxI protein were observed to interact with CAV1 (Figure 7C). This implies that the key interaction domain of ApxI with the host protein CAV1 is mainly located in the acylation region.

## Discussion

*Actinobacillus pleuropneumoniae* is the causative agent of porcine infectious pleuropneumonia, a disease that affects pigs of all ages and has a significant economic and ecological impact on the pig industry. To date, a total of 19 serotypes of *Actinobacillus pleuropneumoniae* strains has been identified [2]. The most important virulence factor of APP is the pore-forming exotoxins family of Apx toxins, including ApxI, ApxII, ApxIII and ApxIV [6]. Despite the presence of Apx toxin-neutralizing antibodies, APP can release Apx toxin directly into the membranes of these cells by adhering to host cells, which leads to the destruction of these cells [19]. Hemolytic and cytotoxic activities are exerted by Apx toxins to varying degrees. ApxI exhibits the greatest cytolytic and hemolytic effects in comparison to other Apx toxins [20]. It has been shown that ApxI toxin produced by APP induces apoptosis in porcine alveolar macrophages [9]. However, little is known about the associated surface membrane proteins involved in the apoptosis of alveolar macrophages induced by ApxI toxin. In this study, we applied the TurboID proximity labeling assay to identify iPAM cellular proteins that interact with ApxI toxin. A total of 318 unique host proteins interacting with ApxI were identified in His-TurboID-ApxIA-treated iPAM cells, and one of these membrane proteins, CAV1, was shown to be involved in ApxI toxin-induced alveolar macrophage apoptosis.

A caveolae is an invagination of the plasma membrane with a diameter of 50–100 nm [21]. During the 1950s, Palade and Yamada discovered caveolae, which play a critical role in the function of mammalian cells [22, 23]. A caveolae consists largely of caveolins, which are protein markers associated with caveolae [24]. The caveolin gene family consists of three members: caveolin-1, caveolin-2, and caveolin-3, of which caveolin-1 plays a significant structural and functional role. Caveolin-1 is widely present in a variety of immune cell types, such as macrophages [25], dendritic cells [26], and mast cells [27].

Previously, it has been shown that many pathogenic microorganisms, including bacteria, viruses, and parasites, rely on endocytosis of caveolae to invade host cells, and this endocytosis is closely related to caveolae protein-1 [28, 29]. Some of these bacterial toxins invade host cells by caveolae-dependent endocytosis. As a pore-forming toxin, aerolysin is produced by *Aeromonas hydrophila* and is capable of concentrating oligomerization on caveolae and forming pores [30].

Basically, His-TurboID-ApxIA and His-TurboID recombinant proteins were expressed and purified before they were identified by SDS‒PAGE and western blot analysis. The mass spectrometry analysis of His-TurboID-ApxIA and His-TurboID-treated iPAM cells identified 1488 and 1315 proteins, respectively. In the His-turboID-ApxIA labeling test, 318 interacting proteins were unique after the background protein was removed. The UniProt website was used to identify the membrane proteins of CAV1 from these 318 host proteins. CAV1 can interact with ApxIA of *A. pleuropneumoniae*, as confirmed by a co-IP assay. Through overexpression and RNA interference studies, we found that CAV1 is involved in the apoptosis of porcine alveolar macrophages induced by ApxI. We identified the key protein for apoptosis of porcine alveolar macrophages induced by ApxI toxin for the first time. However, the role of the CAV1 protein in the pathogenesis of ApxI toxin in vivo remains to be further explored.

In addition, we confirmed that the acylated region of ApxI was the key domain that interacts with CAV1 in porcine alveolar macrophages by constructing different truncated proteins. In 1994, it was found that the RTX family of toxins, which belongs to Apx toxins, interacts with target cells at the initiation site of the acylated structure [31]. Once the acylated structure has established contact with the target cell, insertion of the RTX toxin molecule into the cell membrane requires cell surface protein interactions as well as the action of electrostatic forces. This also corroborates our results.

Using the TurboID proximity labeling system, we have revealed for the first time the proteome of porcine alveolar macrophages interacting with the ApxIA protein of *A. pleuropneumoniae*. We also tentatively established that CAV1 contributes to the apoptosis of porcine alveolar macrophages induced by ApxI toxin. However, the detailed mechanism requires further investigation. This study offers valuable insights that can be utilized to guide prevention, control, and treatment measures for porcine infectious pleuropneumonia, based on the foundational knowledge derived from these research findings.

## Data Availability

Mass spectrometry datasets used and/or analyzed during the current study are available from the corresponding authors upon reasonable request. Other sources of data are publicly available and are identified in the manuscript.
